# Third French Congress on Mesothelioma

**DOI:** 10.1515/pp-2026-0013

**Published:** 2026-07-06

[3^ème^ Journées francophones sur le mésothéliome]

Lyon, April 1^st^ to 2^nd^ 2026

Organizing Committee:

Jean-Baptiste Assié

Nazim Benzerdjeb

Christophe Blanquart

Diane Damotte

Peggy Dartigues

Gabrielle Drevet

Julie Hildt

Didier Jean

Vahan Kepenekian

Sylvie Lantuejoul

Arnaud Scherpereel

Laurent Villeneuve

## Development of potent affitin-based bispecific NK cell engagers for the therapy of MSLN-expressing cancers

Tina Briolay^1*^, Tacien Petithomme^1*^, Hermelyne Gravoueille^2^, Judith Fresquet^1^, Sylvia Lambot^2^, Barbara Mouratou^2^, Agnès Fortun^3,4^, Karine Bernardeau^3^, Agnès Quéméner^1^, Mike Maillasson^1,5^, Erwan Mortier^1,5^, François Davodeau^2^, Frédéric Pécorari^2,§^ and Christophe Blanquart^1,§^

^1^ Nantes Université, Inserm UMR 1307, CNRS UMR 6075, Université d’Angers, CRCI2NA, Nantes, France.

^2^ Nantes Université, Univ Angers, INSERM, CNRS, Immunology and New Concepts in ImmunoTherapy, INCIT, UMR1302/EMR6001, Nantes, France.

^3^ Nantes Université, CHU Nantes, CNRS, Inserm, BioCore, US16, Plateforme P2R, SFR Bonamy, Nantes, France.

^4^ Cibles et Médicaments des Infections et du Cancer, IICiMed, Nantes Université, UR 1155, Nantes, France.

^5^ Nantes Université, Inserm, CNRS, CHU Nantes, SFR Santé, FED 4203, Inserm UMS16, CNRS UMS3556, Imp@ct Platform, Nantes, France.

^*^ These authors have equally contributed to this work as first authors.

^§^ These authors have equally contributed to this work as co-senior authors.

**BACKGROUND**

Well-characterized tumor associated antigens (TAA) represent targets of interest in many anti-cancer therapeutic strategies. In this study, we aimed at developing Affitins directed against human mesothelin (hMSLN). Affitins are a type of small (7 kDa), thermostable and high affinity non-immunoglobulin protein scaffolds derived from Archaea. hMSLN is a TAA overexpressed in pleural mesothelioma (PM) that has been targeted in numerous clinical trials in recent years.

**METHODS**

Ribosome display and next generation sequencing were used to identify hMSLN-specific Affitins. Surface Plasmon Resonance (SPR) and NanoDSF allowed to determine affinity and thermal stability, respectively. hMSLN-expressing and -negative cells were used in 2D and 3D models, under static or flow conditions. Flow cytometry and confocal microscopy were used to characterize Affitins binding properties on cells. NK92hCD16 cells were used to evaluate anti-tumor activity of BiKE on 2D and 3D PM models.

**RESULTS**

We isolated several Affitins specific for hMSLN. The homodimerization of the most promising Affitin (N13) allowed to improve affinity by 60 (from 35 nM to 0.57 nM). This Affitin, monomeric or dimeric, was highly specific and also stable under a wide range of temperatures and upon repeated freeze/thaw cycles. Finally, Bispecific natural killer (NK) cell Engagers (BiKE) composed of an anti-CD16 VHH fused to N13 Affitins (monomeric or dimeric) were constructed. We showed the specific lysis of hMSLN expressing cells in the presence of BiKE and NK cells.

**CONCLUSION**

Thanks to their advantageous properties, Affitins appear as promising affinity agent for the design of BiKEs with potential therapeutic application.

## Stratification of pleural mesothelioma patients for combined immunotherapy based on sarcomatoid component and tumour microenvironment in the IFCT-MAPS2 study

Guillaume Tosato PharmD, PhD^1^, Yuna Blum PhD^2^, Jean-Baptiste Assié MD PhD^3^, Maya Arnould PhD^1^, Clément Meiller, MSc^1^, Marie-Claude Jaurand PhD^1^, Ruth Sequeiros PharmD^4^, Francesca Damiola PhD^4^, Alexandra Langlais MSc^5^, Elodie Amour MSc^5^, Virginie Westeel MD, PhD^6^, Aurélien De Reynies PhD^7^, Julien Mazières MD, PhD^8^, Laurent Greiller MD, PhD^9^, Franck Morin MSc^5^, Sylvie Lantuejoul MD, PhD^10^, Arnaud Scherpereel MD, PhD^11^, Gérard Zalcman MD, PhD^12^, Jean Didier PhD^1^

^1^ INSERM, Université Paris-Cité, Sorbonne Université, Centre de Recherche des Cordeliers, Functional Genomics of Solid Tumours, Paris, France

^2^ Institut de Génétique et Développement de Rennes (IGRD), Université de Rennes, CNRS, INSERM, UMR 6290, ERL U1305, Rennes, France

^3^ INSERM, Université Paris-Cité, Sorbonne Université, Centre de Recherche des Cordeliers, Functional Genomics of Solid Tumours, Paris, France ; Pulmonology department, Centre Hospitalier Intercommunal de Créteil, Université de Paris-Est Créteil, Créteil, France

^4^ Biopathology department, CNR MESOPATH NETMESO MESOBANK, Centre de Lutte Contre le Cancer (CLCC) UNICANCER Léon Bérard, Lyon, France

^5^ Clinical Research Unit, The French Cooperative Thoracic Intergroup (IFCT), Paris, France

^6^ Pneumology department, CHU Besançon, Besançon, France

^7^ INSERM, Université Paris-Cité, Sorbonne Université, Centre de Recherche des Cordeliers, Multi-scale and spatio-temporal modelling in Oncology; Paris, France

^8^ Pulmonology Department, Larrey Hospital, CHU Toulouse, Centre de Recherches en Cancérologie de Toulouse (CRCT), INSERM, CNRS, Université Toulouse III-Paul Sabatier, Toulouse, France

^9^ Department of Multidisciplinary Oncology and Therapeutic Innovations, AP-HM, Université Aix Marseille, Marseille, France

^10^ Biopathology department, CNR MESOPATH NETMESO MESOBANK, Centre de Lutte Contre le Cancer (CLCC) UNICANCER Léon Bérard, Lyon, France; Université Grenoble Alpes, Grenoble, France.

^11^ Pulmonary and Thoracic Oncology department, NETMESO; INSERM U1189 OncoThAI, OncoLille, CHU Lille, Université de Lille, Lille, France

^12^ Thoracic Oncology department, CIC 1425 INSERM, Hôpital Bichat-Claude Bernard, Cancer Institute, AP-HP Nord, Université Paris-Cité, France

**BACKGROUND**

Combination immunotherapy is one of the first-line treatment options in pleural mesothelioma (PM), conferring particular benefit in sarcomatoid PM. However, high-grade adverse events occur in approximately 30% of patients. Transcriptomic and tumor microenvironment (TME) profiling may refine treatment selection and minimize unnecessary toxicity.

**METHODS**

We performed RNA-Seq on tumor samples, obtained at diagnosis, of PM patients from the IFCT-MAPS2 randomized phase II trial comparing nivolumab monotherapy (n=34) versus nivolumab-ipilimumab bitherapy (n=38). The sarcomatoid component was assessed using the transcriptomic S-score, and TME composition was evaluated through deconvolution analysis based on signatures derived from reanalyzed public single-cell RNA-Seq datasets. Survival analyses were conducted using multivariate Cox proportional hazards models adjusted for clinical covariates and IFCT-MAPS2 stratification factors.

**RESULTS**

High S-scores, found in one-third of patients (10 epithelioid, 13 non-epithelioid), interacted with treatment to influence patient outcomes. High S-score patients receiving nivolumab monotherapy had markedly shorter survival than low S-score patients (RR 4.0 [1.7 - 9.5]), whereas no difference was observed in combination therapy (RR 0.6 [0.3 - 1.3]). Deconvolution identified two distinct TME subtypes: CD8-enriched (n=33) and CD4-enriched (n=39). Analysis of immune and stromal cell signatures revealed that some tumor-associated macrophage (TAM) signatures were predictive in a treatment-dependent manner. Indeed, FOLR2.TAM subpopulations, enriched in CD4-dominant tumor subype, correlated with poor survival in nivolumab-treated patients.

**CONCLUSION**

Combination immunotherapy specifically benefits patients with high S-scores and/or elevated TAM subpopulations infiltration. Integrating tumor cell phenotype and TME profiling into clinical decision-making may optimize immunotherapy selection and reduce unnecessary toxicity.

## IMMUNOTHERAPY PLEURAL 5-ALA PDT ‘IMPALA’ Trial: Intrapleural photodynamic therapy via video-assisted thoracoscopy followed by anti-PD-1 immunotherapy (NIVOLUMAB) in patients with malignant pleural mesothelioma

Clément Bouchez^1^, Guillaume Paul Grolez^1^, Bertrand Leroux^1^, Pascal Deleporte^1^, Gregory Baert^1^, Olivier Moralès^1^, Eric Wasielewski^2^, Arnaud Scherpereel^1,2^, Anne-Sophie Dewalle^1^, and Nadira Delhem^1^

^1^ Univ.Lille, Inserm, CHU Lille, U1366-CRCLille – OncoThAI laboratory, 59000 Lille, France.

^2^ Department of Pulmonology and Thoracic Oncology, CHU de Lille, France.

**BACKGROUND**

Mesothelioma is a rare and aggressive cancer, accounting for 80% of all mesothelioma cases. It is often linked to asbestos exposure, which leads to chronic inflammation and cancer. Mesothelioma is more common in men and is often diagnosed at a late stage. Treatment options include surgery, chemotherapy, or immunotherapy. Despite these standard treatments, the average life expectancy ranges from 12 to 13 months. Anti-PD-1 immunotherapy has emerged in recent years as a major treatment for various cancers, including pleural mesothelioma. Photodynamic therapy (PDT) is based on the light-induced activation of a photosensitizer (PS) that accumulates in tumor cells to induce cell death and an immune response. In collaboration between the CHU of Lille and the OncoThAI laboratory, we therefore proposed combining immunotherapy with photodynamic therapy in order to create a synergistic effect between the treatments, thanks to the immune boost provided by PDT. To this end, a novel therapeutic device - the light wand - was developed to illuminate tumorous areas within the pleural cavity.

**METHODS**

The objective of this study is to demonstrate the feasibility and benefits of this treatment combination, without any unacceptable or unexpected toxicity. In addition, immunomonitoring is being conducted to study the patient’s secretome, immunophenotype, and transcriptome throughout the study.

**RESULTS**

To date, patient recruitment is proceeding smoothly; approximately 75% of patients have been enrolled in this trial. Immunomonitoring data for all of these patients is currently being analyzed.

## Tumor-Associated Macrophages Subpopulations Profiling in Pleural Mesothelioma Tumor Samples and Multicellular Tumor Spheroid Models

Guillaume Tosato PharmD, PhD^1^, Tiphaine Delaunay PhD^2^, Maya Arnould PhD^1^, Sophie Deshayes BSc^2^, Clément Meiller MSc^1^, Marie-Claude Jaurand PhD^1^, Jean-Baptiste Assié MD, PhD^3^, Christophe Blanquart PhD^2^, Jean Didier PhD^1^

^1^ INSERM, Université Paris-Cité, Sorbonne Université, Centre de Recherche des Cordeliers, Functional Genomics of Solid Tumours, Paris, France.

^2^ Nantes Université, Inserm UMR 1307, CNRS UMR 6075, Université d’Angers, CRCI2NA, F-44000 Nantes, Nantes, France.

^3^ INSERM, Université Paris-Cité, Sorbonne Université, Centre de Recherche des Cordeliers, Functional Genomics of Solid Tumours, Paris, France; Pulmonology department, Centre Hospitalier Intercommunal de Créteil, Université de Paris-Est Créteil, Créteil, France.

**BACKGROUND**

The pleural mesothelioma (PM) tumor microenvironment (TME) displays substantial intertumoral heterogeneity that is linked to variable responses to combination immunotherapy. The myeloid compartment, which encompasses tumor-associated macrophages (TAM), represents a major immune population in PM and has emerged as potential predictor of immunotherapy sensitivity in other cancers. Understanding TAM diversity may enable better prediction of treatment outcomes as well as patient stratification.

**METHODS**

To reproduce this tumor-driven diversity in preclinical model, we generated multicellular tumor spheroids (MCTS) by co-culturing healthy donor-derived monocytes with 12 distinct PM cell lines. We performed single-nucleus RNA-Seq (snRNA-Seq) on MCTS alongside 15 PM tumor samples to explore TAM subpopulations.

**RESULTS**

Seven TAM subpopulations were identified in PM samples with distinct functional programs: antigen-presenting (PID1.TAM, CD14.TAM, and TREM2.TAM); immunosuppressive (ENTPD1.TAM and CD28.TAM); antitumor (RPS12.TAM and MARCO.TAM). The

proportion of these subpopulations varies greatly from patient to patient, potentially explaining the differences in sensitivity to treatments. Five PM TAM subpopulations were identified in MCTS, with variations dependent on the PM cell line model. TAM phenotypes were reproducible across donors but shaped by the PM cell line, suggesting tumor cell intrinsic signals drive polarization.

**CONCLUSION**

This study establishes a comprehensive myeloid landscape map in PM and highlight that tumor cells drive TAM polarization. We highlight MCTS as a relevant preclinical model for characterizing TAM subpopulations and developing tailored therapeutic strategies targeting their heterogeneity to improve immunotherapy outcomes in PM.

## New photodynamic therapy strategy using a third-generation photosensitizer to target neuropilin-1 in pleural mesothelioma

Clément Bouchez^1*^, Kimberley Pridannikoff^1*^, Morgane Moinard^2^, Guillaume Paul Grolez^1^, Pascal Deleporte^1^, Grégory Baert^1^, Anne-Sophie Dewalle^1^, Samir Acherar^2^, Céline Frochot^2^, Arnaud Scherpereel^1,3^, Nadira Delhem^1^ and Olivier Moralès^1^

^1^ Univ.Lille, Inserm, CHU Lille, U1366-CRCLille – OncoThAI laboratory, 59000 Lille, France.

^2^ CNRS UMR 7274 – LRGP team, Université de Lorraine, Nancy, France.

^3^ Department of Pulmonology and Thoracic Oncology, CHU de Lille, France.

* These authors have equally contributed to this work as first authors.

**BACKGROUND**

Pleural mesothelioma is often linked to asbestos exposure, leading to chronic inflammation and cancer. Despite treatment, life expectancy is 13 months. Photodynamic therapy (PDT) is based on the light-induced activation of a photosensitizer (PS) that accumulates in tumor cells to induce cell death and an immune response. Neuropilin-1 (NRP1) is a biomarker that is overexpressed in mesothelioma cells, tumor-infiltrating regulatory T cells (Tregs), and tumor endothelial cells. We have designed a PS targeting NRP-1 that enables tumor destruction accompanied by an anti-angiogenic effect, while reducing immunosuppression and enhancing the immune response.

**METHODS**

The PS was characterized using photophysical and photochemical assays. NRP-1 expression was assessed at the transcriptomic and proteomic levels. The affinity of the PS was determined by micro-scale thermophoresis, and PS incorporation into cells was validated using fluorescence imaging. The efficacy of PDT was demonstrated by measuring cell viability, validated by the production of reactive oxygen species and the type of cell death induced, with no toxicity from the unactivated PS. To corroborate the results from endothelial cells, we demonstrated the efficacy of PDT on blood vessels on chips.

**RESULTS**

We have demonstrated that the synthesized PS has excellent properties and that NRP-1 is expressed in our models. The PS binds specifically to NRP-1 to enter cells in a dose- and time-dependent manner. PDT is effective in all three cell types without being toxic outside of photoactivation. This efficacy depends on ROS production and results in significant immunogenic cell death.

**CONCLUSION**

Our PS could enable specific and effective antitumor PDT through the significant production of ROS, leading to potential immunogenic necrotic cell death that could trigger a specific immune response.

## Surgical management of peritoneal mesothelioma

Vahan Kepenekian MD, PhD^1,2^, Cécile Odin^1,2^, Claire-Angeline Goutard^1,2^, Nazim Benzerdjeb MD, PhD^2,3^, Julien Péron MD, PhD^4^, Pascal Rousset MD, PhD^2,5^, Olivier Glehen MD, PhD^1,2^, Laurent Villeneuve PhD^2,6^

^1^ Surgical Oncology Department, Hôpital Lyon Sud, Hospices Civils de Lyon (HCL), Pierre Bénite, France.

^2^ Université Lyon 1, Hospices Civils de Lyon, CICLY, EA 3738, Lyon, France.

^3^ Pathology Department, Hôpital Lyon Sud, Hospices Civils de Lyon (HCL), Pierre Bénite, France.

^4^ Medical Oncology Department, Hôpital Lyon Sud, Hospices Civils de Lyon, Lyon, France; Université Claude Bernard Lyon I (UCBL1), Laboratoire de Biométrie et Biologie Evolutive, Equipe Biostatistique-Santé, Lyon, France.

^5^ Radiology Department, Hôpital Lyon Sud, Hospices Civils de Lyon, EA3738, CICLY, Centre pour l’Innovation en cancérologie de Lyon, Université Claude Bernard Lyon I (UCBL1), Lyon, France.

^6^ Clinical Research Department, Hospices Civils de Lyon, Lyon, France.

Peritoneal mesothelioma is a rare disease (1–3 cases/million/year) with predominantly locoregional spread, which makes it amenable to peritoneal-directed therapies. Its prognosis is thus largely determined by the ability to achieve complete cytoreductive surgery (CRS) typically followed by hyperthermic intraperitoneal chemotherapy (HIPEC).

The surgical strategy is built around two pillars: resectability and operability. Resectability assessment requires defining the Peritoneal Cancer Index (PCI), in particular the extent of small bowel involvement and the expected digestive resections to achieve complete cytoreduction. Staging laparoscopy plays a central role in this evaluation. Operability relies on a comprehensive multimodal assessment, including oncogeriatric evaluation where appropriate.

CRS follows the radical principles described by Sugarbaker, combining peritonectomy procedures with organ resections. Perioperative management - including prehabilitation and enhanced recovery protocols - is essential to limit severe morbidity, which remains significant despite improvements over time. Complications have been shown to negatively impact long-term survival.

Long-term results from the RENAPE registry (selected 288 epithelioid mesotheliomas with long-term follow-up and CC-0/1 CRS-HIPEC) demonstrate an estimated 10-year recurrence-free survival of 28% with a median of 37 months. Even patients with high PCI or CC-1 resections could achieve prolonged survival. In selected patients, iterative CRS yields equivalent outcomes when feasible.

However, half of patients are not resectable at diagnosis. Bidirectional strategies combining neoadjuvant systemic and intraperitoneal chemotherapy (NIPEC, PIPAC) enable conversion to complete resection in up to 50% of initially unresectable cases.

Early referral to an expert center dedicated to peritoneal malignancies remains paramount for optimal multidisciplinary management aiming at bringing patients to complete cytoreduction.

## The French Network for Rare Peritoneal Cancers (RENAPE)

Laurent Villeneuve PhD^1,2^, Cécile Odin^2,3^, Vahan Kepenekian MD, PhD^2,3^, Nazim Benzerdjeb MD, PhD^2,4^, Peggy Dartigues MD^5^, Frédéric Bibeau MD, PhD^6^, Julien Péron MD, PhD^7^, Pascal Rousset MD, PhD^2,8^, Olivier Glehen MD, PhD^2,3^

^1^ Department of Clinical Research, Hospices Civils de Lyon, Lyon, France.

^2^ Université Lyon 1, Hospices Civils de Lyon, CICLY, EA 3738, Lyon, France.

^3^ Department of Digestive and Oncological Surgery, Hôpital Lyon Sud, Hospices Civils de Lyon, Lyon, France.

^4^ Department of Pathology, Hôpital Lyon Sud, Hospices Civils de Lyon, Lyon, France.

^5^ Medical Biology and Pathology Department, Pathology Laboratory, Gustave Roussy, University of Paris-Saclay, Villejuif, France.

^6^ Department of Pathology, Besançon University Hospital, Besançon, France.

^7^ Institut de Cancérologie des Hospices Civils de Lyon, Lyon, France.

^8^ Department of Radiology, Hôpital Lyon Sud, Hospices Civils de Lyon, Lyon, France.

**BACKGROUND**

Rare peritoneal cancers such as mesothelioma and pseudomyxoma peritonei, present significant diagnostic and therapeutic challenges. The RENAPE network was established to address these issues by standardizing care, reducing treatment delays, and improving patient outcomes through multidisciplinary collaboration and specialized expertise.

**METHODS**

RENAPE operates through a hub-and-spoke model connecting expert centers and regional tumor boards. Key initiatives included double-reading pathology reviews (RENA-PATH) for diagnostic accuracy, standardized imaging protocols (RENA-RAD) for disease assessment, and specialized education programs. The network also advances clinical research through clinical and translational studies.

**RESULTS**

Since 2011, RENAPE has achieved major progress in the management of rare peritoneal cancers. The network has reduced median treatment delays from 4.73 months to 2.35 months, ensuring patients receive timely care. Over 80 complex cases are reviewed annually by expert pathology panels, improving diagnostic precision and guiding personalized treatment strategies. RENAPE has also edited national and international clinical guidelines, setting standards for best clinical practices for diagnosis and therapy. The network has implemented a national registry with over 5,150 cases. Additionally, RENAPE collaborates actively with the patient advocacy group (AMARAPE) to integrate patient-reported experiences and perspectives into continuous improvements in care and research.

**CONCLUSION**

RENAPE has significantly improved the management of rare peritoneal cancers in France by standardizing care, enhancing diagnostic accuracy, and promoting research. Its structured approach ensures better patient outcomes and continued progress in addressing these complex diseases.

## Subclonal selection as determinant of sarcomatoid progression in pleural mesothelioma

Maya Arnould PhD^1^, Jean-Baptiste Assié MD, PhD^1,2^, Clément Meiller MSc^1^, Ruth Sequeiros MSc^3^, Sandrine Imbeaud PhD^1^, Amélie Roehrig PhD^1^, Camille Boano MSc^1^, Delphine Le Corre^4^, Sophie Mouillet-Richard PhD^4^, Lucie Poupel PhD^5^, Julien Lavergne^5,6^, Véronique Hofman MD, PhD^7^, Paul Hofman MD, PhD^7^, Sarah Humez MD^8,9^, Arnaud Scherpereel MD, PhD^10,11^, Françoise Le Pimpec-Barthes MD, PhD^1,12,13^, Jessica Zucman-Rossi MD, PhD^1^, Christophe Blanquart PhD^14^, Marie-Claude Jaurand PhD^1^, Sylvie Lantuejoul MD, PhD^3,15^, Theo Z Hirsch PhD^1^, Diane Damotte MD, PhD^16,17^, Didier Jean PhD^1^

^1^ Centre de Recherche des Cordeliers, INSERM, Université Paris Cité, Sorbonne Université, Functional Genomics of Solid Tumors, Paris, France.

^2^ Centre Hospitalier Intercommunal de Créteil, Université de Paris-Est Créteil, Department of Pneumology, Créteil, France.

^3^ Centre Léon Bérard, Cancer Research Center of Lyon, Reference National Center MESOPATH and MESOBANK, Department of BioPathology, Lyon, France.

^4^ Centre de Recherche des Cordeliers, INSERM, Université Paris Cité, Sorbonne Université, Personalized medicine, pharmacogenomics, therapeutic optimization team, Equipe labellisée Ligue Nationale Contre le Cancer, Paris, France.

^5^ Centre de Recherche des Cordeliers, INSERM, Université Paris Cité, Sorbonne Université Cordeliers Histology Imaging Cytometry & Spatial-omics (CHICS) Paris, France.

^6^ AP-HP.Centre-Université Paris Cité, Institut du Cancer Paris CARPEM, Paris, France.

^7^ IHU RespirERA, FHU OncoAge, Biobank BB-0033-00025, Inserm U1081, Université Côte d’Azur, Nice, France.

^8^ Univ. Lille, CHU Lille, Institut de Pathologie, Lille, France

^9^ Univ. Lille, CNRS, Inserm, CHU Lille, Institut Pasteur de Lille, UMR9020 – UMR1277 - Canther – Cancer Heterogeneity, Plasticity and Resistance to Therapies, Lille, France

^10^ Univ. Lille, CHU Lille, Service de Pneumologie et d’Oncologie Thoracique, unité INSERM 1189 OncoThAI, Lille, France

^11^ Réseau National Expert pour le Mésothéliome Pleural Malin (NETMESO), Lille, France

^12^ Assistance Publique-Hôpitaux de Paris, Hôpital Européen Georges Pompidou, Paris, France

^13^ Service de Chirurgie Thoracique, Hôpital Européen Georges Pompidou, Paris, France

^14^ Nantes Université, Inserm UMR 1307, CNRS UMR 6075, Université d’Angers, CRCI2NA, F-44000 Nantes, France.

^15^ Université Grenoble Alpes, Grenoble, France.

^16^ Centre de Recherche des Cordeliers, INSERM, Université Paris Cité, Sorbonne Université Inflammation, Complement and Cancer Team, Paris, France.

^17^ Service de Pathologie, Hôpital Cochin, Assistance Publique Hôpitaux de Paris Centre, Université Paris Cité, Paris, France.

**BACKGROUND**

A better understanding of intratumoral heterogeneity in pleural mesothelioma (PM) is essential for implementing precision medicine for this cancer. This heterogeneity remains understudied, particularly with regard to the clonal evolution of tumor cells.

**METHODS**

We performed single-cell and single-nucleus RNA-seq on 15 tumor samples and 14 primary cell lines derived from patients, respectively. The samples were selected to be representative of intertumor heterogeneity in terms of commitment to an epithelioid or sarcomatoid phenotype and mutational profile. Both transcriptomic profiles and copy number variations (CNVs), were analyzed using inference tools such as inferCNV and Numbatfor each tumor cell.

**RESULTS**

Transcriptomic analyses allowed us to define four phenotypic cell types: epithelioid, sarcomatoid, intermediate, and undifferentiated. By exploring the presence of these phenotypic cell types in RNA-seq data from larger patient cohorts, we demonstrated that they were associated with overall survival. Some tumor samples also exhibited intratumoral phenotypic heterogeneity, with the presence of multiple phenotypic cell types. Interestingly, this heterogeneity was consistently associated with subclonal evolution, in which sarcomatoid cells emerged from parental cells exhibiting an intermediate phenotype following the acquisition of CNVs. The subclones were maintained in cell lines and retained their specific phenotypes, suggesting high phenotypic heritability. A spatial transcriptomic analysis validated the link between phenotypic and subclonal evolution, showing that subclonal tumor cells were colocalized within the tumor tissue.

**CONCLUSION**

Our study provides a comprehensive single-cell profiling of PM tumor cells and reveals a close link between phenotypic evolution, clonal architecture, and the spatial organization of the tumor.

## Systemic treatments in peritoneal mesothelioma: recommendations, current practices and perspectives

Julien Péron, MD, PhD^1^

^1^Institut de Cancérologie des Hospices Civils de Lyon, Lyon, France

**BACKGROUND**

Peritoneal mesothelioma (PM) is a rare and aggressive malignancy with limited systemic treatment options. While cytoreductive surgery with hyperthermic intraperitoneal chemotherapy (HIPEC) remains the cornerstone of management, systemic therapy is required in patients with unresectable or relapsed disease. The current therapeutic landscape is characterized by a single established first-line standard and an unmet need for effective subsequent therapies.

**METHODS**

This review synthesizes current evidence and expert recommendations regarding systemic therapies for PM, drawing on published clinical trials, recent congress data (ASCO, ESMO, and PSOGI 2024–2025), and a French multicenter cohort (Péron et al., in press). Emerging strategies including immunotherapy combinations, CAR-T cell therapies, cancer vaccines, and targeted agents (YAP/TEAD inhibitors) are reviewed.

**RESULTS**

First-line platinum-pemetrexed chemotherapy remains the only validated standard, yielding a median OS of 38.9 months in PM (n=139). Second-line outcomes are poor (mOS ∼14.9 months, mPFS ∼3 months). Immunotherapy combinations show proof-of-concept activity (ORR 19–75%), though data in PM specifically remain limited. The IMMUNORARE trial is prospectively evaluating domvanalimab + zimberelimab in PM and other rare tumors. CAR-T cells targeting mesothelin (Gavo-cell) show encouraging early results. YAP/TEAD inhibitors demonstrate preliminary efficacy in mesothelioma in phase 1 and 2 studies.

**CONCLUSION**

Despite emerging therapeutic avenues, prognosis in peritoneal mesothelioma remains primarily driven by surgical eligibility. Immunotherapy and novel targeted agents represent promising investigational strategies, but prospective data in PM are still needed to establish new standards of care.

## Development of a vascularized model of pleural mesothelioma to study oncolytic activity of the vaccinal strain of measle virus

Nikita Rajkumari^1^, Mégane Willems^1^, Judith Fresquet^1^, Elise Douillard^1^, Magali Devic^1^, Hortense Perdrieau^1^, Delphine Fradin^1^, Jean-François Fonteneau^1^, Nicolas Boisgerault^1^, Isabelle Corre^1^, Lucas Treps^1^, Boudewijn Van der Sanden^2,*^ and Christophe Blanquart^1,*^

^1^ Nantes Université, Inserm UMR 1307, CNRS UMR 6075, Université d’Angers, CRCI2NA, Nantes, France.

^2^ Univ. Grenoble Alpes, CNRS, TIMC-IMAG UMR 5525, La Tronche, France.

^*^ These authors have equally contributed to this work as co-senior authors.

**BACKGROUND**

Pleural mesothelioma (PM) is a rare and aggressive cancer mainly caused by asbestos exposure. Although dual immune checkpoint blockade (anti-PD-1/anti-CTLA-4) is approved as first-line therapy, its clinical benefit remains limited. We previously demonstrated that the attenuated Schwarz strain of measles virus (MV) exhibits oncolytic activity against PM cells. However, preclinical evaluation is hindered by the non-permissiveness of rodent models for MV. To address this limitation, we developed a humanized 3D “vascularized mesothelioma-on-chip” (VMOC) model using microfluidic technology.

**METHODS**

Commercial IdenTX microfluidic chips (AIM Biotech) were used. The model consists of two perfusable endothelial-lined vessels flanking a central microvascular network (MVN), generated using human umbilical vein endothelial cells (HUVECs) embedded in fibrin and cocultured with PM cells and primary human lung fibroblasts (hLFs). The model and MV oncolytic activity were characterized by confocal microscopy, multiplex ELISA, single-cell RNA sequencing, 3’ RNA sequencing, and luminescent assays.

**RESULTS**

We confirmed the presence of adherens and tight junctions within the endothelial compartment. Injection of 70 kDa FITC-dextran demonstrated functional connectivity between parental vessels and the MVN. Distinct cell subpopulations, resembling those observed in vivo, were identified across all cell types in the VMOC. Following MV administration through the endothelial network, PM cell infection and death were observed, along with strong activation of the type I interferon pathway and increased production of inflammatory mediators.

**CONCLUSION**

The VMOC model enables in vitro investigation of MV infection and tumor microenvironment reprogramming, providing a valuable platform to better understand treatment responses.

## Dissecting TEAD dependency in pleural mesothelioma: targeting TEAD1 is required but not sufficient

Khawla Bennani Ziatni PhD^1,2^, Clément Meiller MSc^1^, Maya Arnould PhD^1^, Laurent Dassencourt MSc^2^, Odette Dos Santos MSc^2^, Armelle Buzy PhD^3^, Françoise Le Pimpec-Barthes MD, PhD^1,4^, Jean-Baptiste Assié MD, PhD^1,5^, Iris Valtingojer PhD^2^, Jean-Christophe Le Bail PhD^2^, Didier Jean PhD^1^

^1^ Centre de Recherche des Cordeliers, INSERM, Université Paris Cité, Sorbonne Université, Functional Genomics of Solid Tumors, Paris, France.

^2^ Oncology, Sanofi R&D, Sanofi, Vitry-sur-Seine, France.

^3^ Structural Biology, Sanofi R&D, Sanofi, Vitry-sur-Seine, France.

^4^ Department of Thoracic Surgery, Hôpital Européen Georges Pompidou, Assistance Publique Hôpitaux de Paris, Paris, France.

^5^ Department of Pneumology, Centre Hospitalier Intercommunal de Créteil, Université de Paris-Est Créteil, Créteil, France.

**BACKGROUND**

The Hippo signaling pathway is frequently dysregulated in pleural mesothelioma (PM), contributing significantly to tumor progression. The downstream effector of this pathway, the TEAD-YAP/TAZ transcriptional complex, is a highly promising therapeutic target, particularly the TEAD transcription factors, which are targetable with available inhibitors. However, the respective roles and contributions of the four TEAD paralogs (TEAD1/4) in PM remain poorly understood.

**METHODS**

We assessed TEAD gene expression in four independent PM cohorts. Functional studies were conducted on a large series of well-characterized patient-derived primary PM cell lines, representative of tumor heterogeneity. Seven TEAD inhibitors with defined TEAD1/4 specificities were screened for cell viability. TEAD1/4 contribution was further evaluated by RNA interference-based combined silencing and gene dependency dataset analysis. RNA-seq profiling was performed to characterize downstream pathway modulation upon TEAD combined knockdown.

**RESULTS**

TEAD1 was the most highly expressed TEAD family member across all cohorts. This over-representation is conserved at the protein level in cell lines, whereas TEAD2 protein expression is low. TEAD inhibitors markedly reduced cell viability independently of Hippo pathway mutation status or YAP/TAZ-TEAD activation. TEAD1 emerged as the primary contributor to cell viability. However, effective inhibition required simultaneous targeting of TEAD1, TEAD3 and TEAD4. Transcriptomic analysis revealed impacts on cell cycle programs and activation of inflammatory and interferon signaling pathways.

**CONCLUSION**

Our results clarify the contribution of TEAD1/4 paralogs in PM, reinforce the therapeutic relevance of TEAD inhibition, and provide valuable insights for the development of future therapeutic strategies targeting TEAD in this cancer.

